# Low on-treatment diastolic blood pressure and cardiovascular outcome: A post-hoc analysis using NHLBI SPRINT Research Materials

**DOI:** 10.1038/s41598-019-49557-4

**Published:** 2019-09-10

**Authors:** Piotr Sobieraj, Jacek Lewandowski, Maciej Siński, Zbigniew Gaciong

**Affiliations:** Department of Internal Medicine, Hypertension and Vascular Diseases, The Medical University of Warsaw, Warsaw, 02-091 Poland

**Keywords:** Hypertension, Vascular diseases

## Abstract

Recent studies including the SPRINT trial have shown beneficial effects of intensive systolic blood pressure reduction over the standard approach. The awareness of the J-curve for diastolic blood pressure (DBP) causes some uncertainty regarding the net clinical effects of blood pressure reduction. The current analysis was performed to investigate effects of low on-treatment DBP on cardiovascular risk in the SPRINT population. The primary composite outcome was the occurrence of myocardial infarction, acute coronary syndrome not resulting in myocardial infarction, stroke, acute decompensated heart failure or death from cardiovascular causes. The prevalence of primary outcomes was significantly higher in subjects within low DBP in both standard (44–67 mmHg [10.8%] vs 67–73 mmHg [6.7%] vs 73–78 mmHg [5.1%] vs 78–83 mmHg [4.4%] vs 83–113 mmHg [4.3%], p < 0.001) and intensive treatment (38–61 mmHg [6.7%] vs 61–66 mmHg [4.1%] vs 66–70 mmHg [4.5%] vs 70–74 mmHg [2.7%] vs 74–113 mmHg [3.4%], p < 0.001) arms. After adjusting for covariates, low DBP showed no significant effects on cardiovascular risk. Therefore, while reaching blood pressure targets, low DBP should not be a matter of concern.

## Introduction

The American College of Cardiology, American Heart Association and the European Society of Cardiology, recently adopted new goals for antihypertensive therapy following the release of results from new clinical studies including the Systolic Blood Pressure Intervention Trial (SPRINT)^1–3^. SPRINT showed that achieving systolic blood pressure (SBP) of less than 120 mmHg, rather than 140 mmHg, contributed to reduced risks of fatal and nonfatal major cardiovascular events and overall mortality. The new recommendations supporting intensive blood pressure reduction meet the fact that a significant percentage of patients receiving antihypertensive therapy still do not achieve their target BP values^4^. While lower BP reduced the risk of cardiovascular events and improved survival, it also created doubts regarding the J-curve hypothesis. In 1979 it was shown that lower diastolic blood pressure (DBP) may increase the risk of myocardial infarction among hypertensive patients^5,6^. The results of other studies addressing this issue confirmed that intensive DBP reduction may increase the risk of coronary artery disease (CAD)^7,8^. There are few studies published before and after SPRINT which established an optimal target DBP. Randomised trials showed inconclusive results or considered out-dated DBP targets^9,10^. Other investigations comprised post-hoc analyses of randomised trial participants, including the SPRINT population^11–15^. Overall, the results of these studies were incoherent, applied methodologies differed between studies and investigators pooled data from different studies, such as one report which combined data from SPRINT and the Action to Control Cardiovascular Risk in Type 2 Diabetes (ACCORD). The International Verapamil–Trandolapril Study (INVEST) study focused on hypertensive subjects with stable CAD and J-curve relationships between both systolic and diastolic BP and primary outcomes were found^16^. However, the J- shape of the curve was more pronounced for DBP than for SBP, and the risk of myocardial infarction and death increased when the DBP was <75 mmHg^16^. Considering these findings, it is still unclear whether a substantial BP reduction is beneficial and safe among all subgroups of hypertensive patients. In the SPRINT study, intensive control of SBP reduced the risk of cardiovascular disease among high-risk patients^1^. In contrast, the ACCORD study, which included diabetic patients, demonstrated that a similar reduction in SBP did not affect cardiovascular morbidity and mortality^17^. In this study, we evaluated the effects of low on-treatment DBP on primary composite outcomes and investigated whether other clinical factors influenced these outcomes in hypertensive subjects.

## Results

A total of 8890 participants (64.7% men, 35.3% women) were included in this study. Among these participants, 4438 (49.9%) of them were allocated to the standard (<140 mmHg) treatment arm while 4452 (50.1%) patients were placed in the intensive treatment arm (<120 mmHg). The primary composite outcome occurred a total of 461 (5.2%) subjects, including 272 (6.1%) from the standard and 189 (4.2%) from the intensive treatment arm. The overall mean SBP/DBP was 128.1/71.4 mmHg, while mean SBP/DBP in standard and intensive treatment arm were 135.3/75.1 mmHg and 120.9/67.7 mmHg.

**Figure 1:**
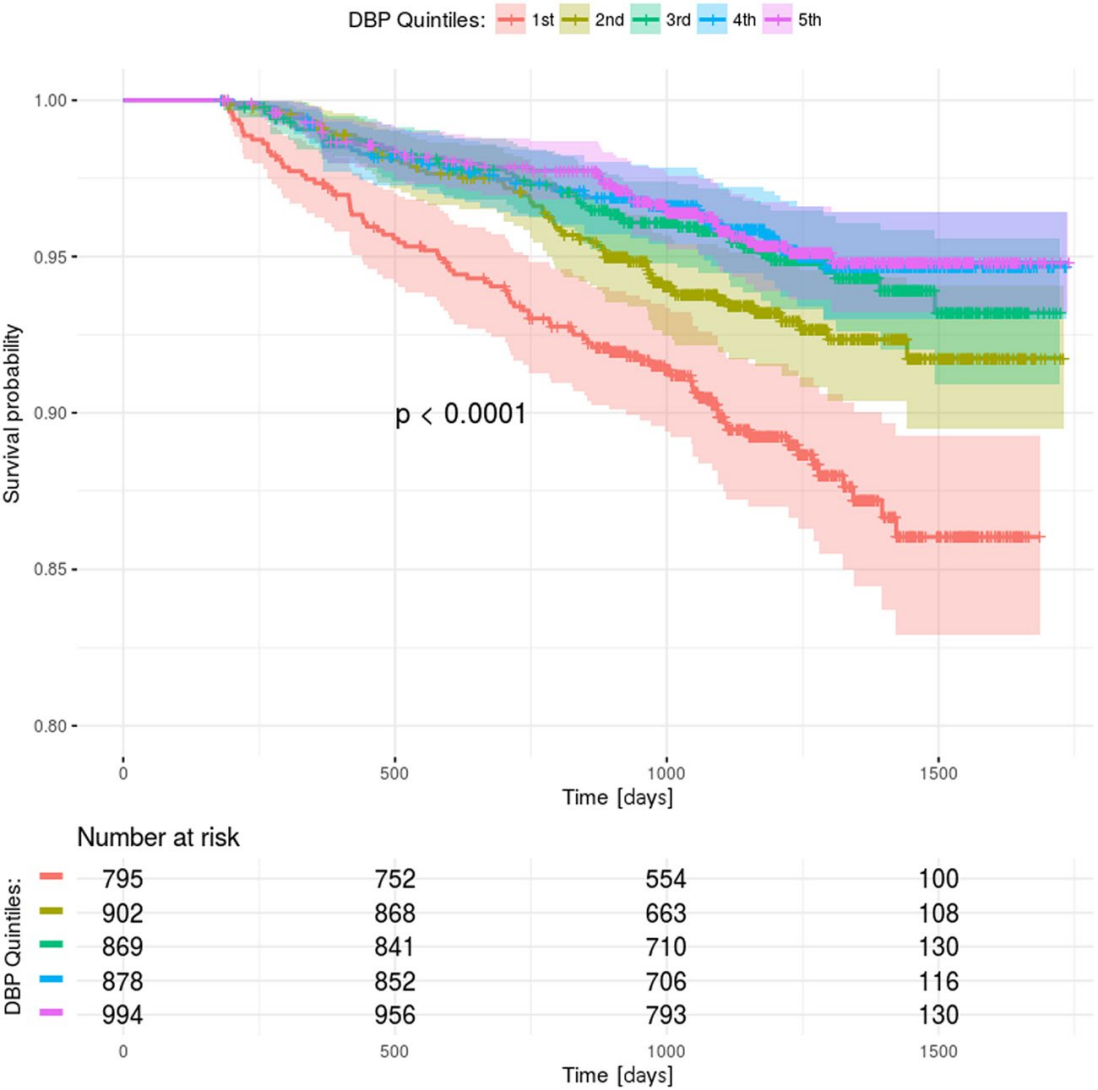
Kaplan-Meier curves with 95% confidence intervals in standard treatment arm. Comparison of curves within DBP quintiles were made using the log-rank test (p < 0.0001). 1st quintile: 44–67 mmHg, 2nd quintile: 67–73 mmHg, 3rd quintile: 73–78 mmHg, 4th quintile: 78–83 mmHg, 5th quintile: 83–113 mmHg.

**Figure 2:**
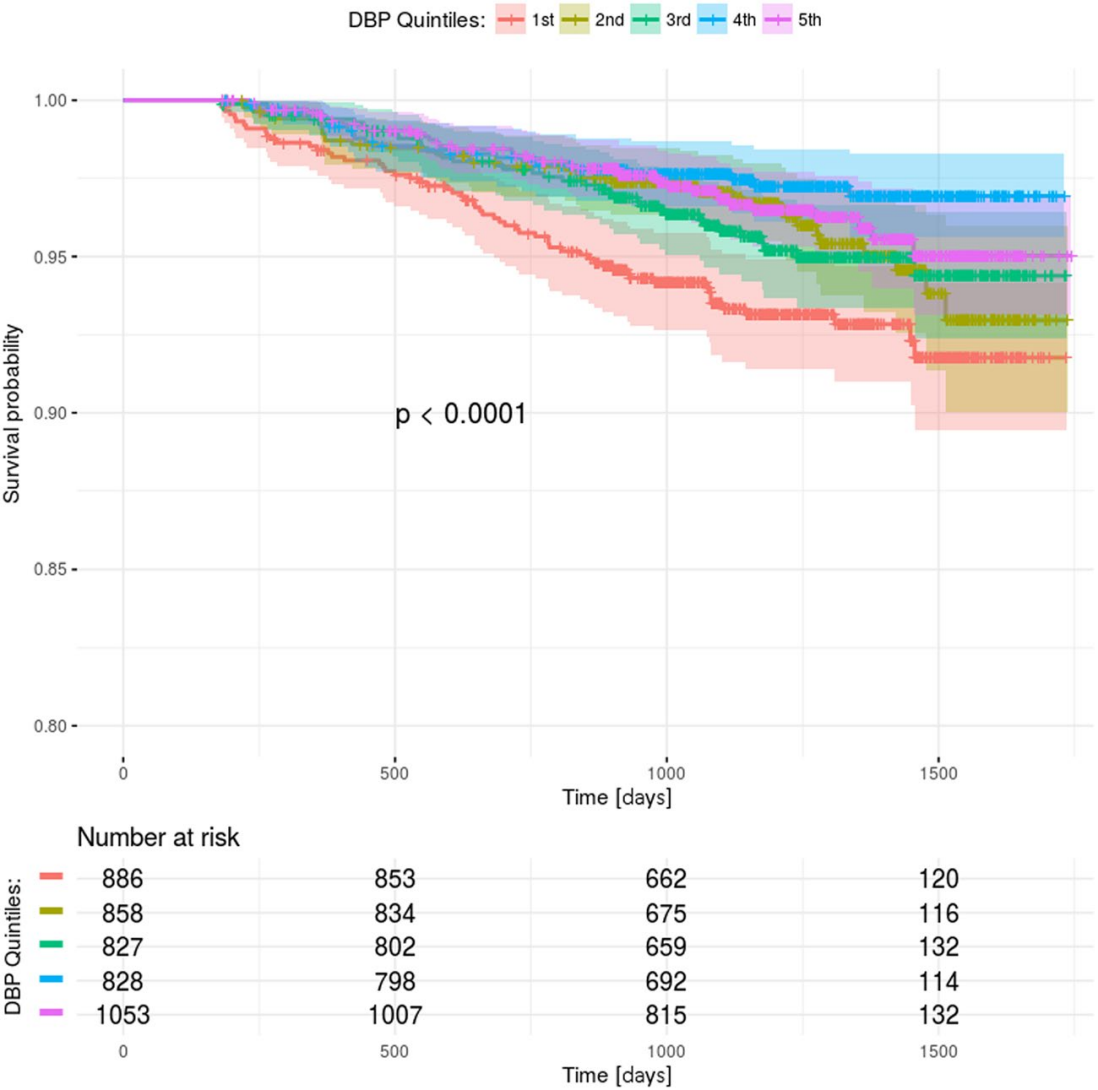
Kaplan-Meier curves with 95% confidence intervals in intensive treatment arm. Comparison of curves within DBP quintiles were made using the log-rank test (p < 0.0001). 1st quintile: 38–61 mmHg, 2nd quintile: 61–66 mmHg, 3rd quintile: 66–70 mmHg, 4th quintile: 70–74 mmHg, 5th quintile: 74–113 mmHg.

The quintiles of DBP were calculated for the standard (1^st^ quintile 44–67 mmHg, 2^nd^ 67–73 mmHg, 3^rd^ 73–78 mmHg, 4^th^ 78–83 mmHg, 5^th^ 83–113 mmHg) and intensive treatment arm (1^st^ quintile 38–61 mmHg, 2^nd^ 61–66 mmHg, 3^rd^ 66–70 mmHg, 4^th^ 70–74 mmHg, 5^th^ 74–113 mmHg). There were significant differences in the occurrence of primary endpoints within quintiles of both treatment arms (standard: 10.8% vs 6.7% vs 5.1% vs 4.4% vs 4.3%, p < 0.001; intensive: 6.7% vs 4.1% vs 4.5% vs 2.7% vs 3.4%, p < 0.001). Kaplan–Meier curves showing survival rates among the arms are presented in Figs 1 and 2. Log-rank test confirmed significant differences between curves among the DBP quintiles in the standard and intensive treatment arms (p < 0.0001). The clinical characteristics of the quintiles in each treatment arm are shown in Tables 1 and 2.

**Table 1:** Characteristics of DBP quintiles within the standard treatment arm. Continuous data are presented as mean ± standard deviation, discrete as a number with percentage.

Parameter	Subjects in standard treatment arm N = 4438	1st quintile 44–67 mm Hg N = 795	2nd quintile 67–73 mm Hg N = 902	3rd quintile 73–78 mm Hg N = 869	4th quintile 78–83 mm Hg N = 878	5th quintile 83–113 mm Hg N = 994	p-value
Primary endpoint event (%)	272 (6.1)	86 (10.8)	60 (6.7)	44 (5.1)	39 (4.4)	43 (4.3)	<0.001
Time to event/censoring [days]	1174.5 ± 286.6	1133.6 ± 307.3	1156.8 ± 283.6	1204 ± 284.3	1198.1 ± 272.6	1176.6 ± 281.8	<0.001
Age [years]	67.8 ± 9.4	76.2 ± 7.3	71.8 ± 8.3	67.6 ± 8.1	64.7 ± 7.5	60.5 ± 7	<0.001
On-treatment DBP [mm Hg]	75.1 ± 9.2	61.1 ± 4.3	69.7 ± 1.7	75 ± 1.4	79.9 ± 1.4	86.9 ± 3.6	<0.001
On-treatment SBP [mm Hg]	135.3 ± 7.3	133.7 ± 8.9	134.4 ± 7.3	135.2 ± 6.8	135.8 ± 6	137.2 ± 7	<0.001
On-treatment PP [mm Hg]	60.2 ± 10.6	72.5 ± 9.7	64.7 ± 7.5	60.1 ± 6.9	55.9 ± 6.1	50.4 ± 6.6	<0.001
Baseline DBP [mm Hg]	78.1 ± 11.9	65.4 ± 8.5	73.3 ± 8.3	78 ± 8.5	82.8 ± 9.3	88.5 ± 9.9	<0.001
Baseline SBP [mm Hg]	139.7 ± 15.4	140.6 ± 16	139.2 ± 15	139.1 ± 15.4	139.9 ± 15.7	139.5 ± 14.7	0.343
Baseline PP [mm Hg]	61.6 ± 14.3	75.2 ± 14.6	66 ± 11.8	61.2 ± 11.6	57 ± 11	51 ± 9.8	<0.001
Female (%)	1545 (34.8)	297 (37.4)	354 (39.2)	317 (36.5)	269 (30.6)	308 (31)	<0.001
Black race (%)	1394 (31.4)	139 (17.5)	211 (23.4)	246 (28.3)	332 (37.8)	466 (46.9)	<0.001
History of cardiovascular disease (%)	733 (16.5)	215 (27)	186 (20.6)	126 (14.5)	120 (13.7)	86 (8.7)	<0.001
History of chronic renal disease (%)	1228 (27.7)	335 (42.1)	289 (32)	222 (25.5)	190 (21.6)	192 (19.3)	<0.001
Body mass index [kg/m^2^]	29.8 ± 5.7	27.8 ± 4.9	29 ± 5.5	30.1 ± 5.7	30.6 ± 5.8	31.2 ± 5.8	<0.001
Current smoking (%)	566 (12.8)	54 (6.8)	59 (6.5)	96 (11)	130 (14.8)	227 (22.8)	<0.001
Former smoking (%)	1901 (42.8)	412 (51.8)	419 (46.5)	378 (43.5)	362 (41.2)	330 (33.2)	<0.001
Never smoking (%)	1965 (44.3)	328 (41.3)	423 (46.9)	394 (45.3)	386 (44)	434 (43.7)	0.197
Number of antihypertensive drugs	1.8 ± 1	2 ± 1.1	1.9 ± 1	1.8 ± 1	1.7 ± 1	1.8 ± 1.1	<0.001
Statin (%)	1972 (44.8)	442 (56)	468 (52.3)	385 (44.6)	361 (41.3)	316 (32.1)	<0.001
Aspirin (%)	2244 (50.7)	481 (60.7)	504 (55.9)	446 (51.4)	419 (47.7)	394 (39.8)	<0.001
Cholesterol [mg/dl]	189.9 ± 40.5	179.8 ± 38.3	186 ± 41.5	191.3 ± 40.5	193.6 ± 39.7	197 ± 40.3	<0.001
HDL [mg/dl]	52.8 ± 14.6	53.6 ± 14.3	54.6 ± 15.2	53 ± 14.7	51.5 ± 14.6	51.4 ± 13.7	<0.001
Non-HDL [mg/dl]	137.1 ± 38.5	126.2 ± 35.9	131.4 ± 38.8	138.4 ± 38.4	142.1 ± 37	145.6 ± 38.6	<0.001
Triglyceride [mg/dl]	126.9 ± 94.3	119.3 ± 71.2	120.1 ± 127.1	125.8 ± 71.4	134.2 ± 92.2	133.8 ± 94.4	<0.001
Glucose [mg/dl]	98.8 ± 13.3	99.6 ± 12.7	98 ± 11.9	98.9 ± 12.3	99.2 ± 12.9	98.2 ± 15.8	0.281

SBP – systolic blood pressure, DBP – diastolic blood pressure, PP – pulse pressure, p-value calculated for comparison within quintiles.

**Table 2:** Characteristic of DBP quintiles within the intensive treatment arm. Continuous data are presented as mean ± standard deviation, discrete as a number with percentage.

Parameter	Subjects in intensive treatment arm N = 4452	1st quintile 38–61 mm Hg N = 886	2nd quintile 61–66 mm Hg N = 858	3rd quintile 66–70 mm Hg N = 827	4th quintile 70–74 mm Hg N = 828	5th quintile 74–113 mm Hg N = 1053	p-value
Primary endpoint event (%)	189 (4.3)	59 (6.7)	35 (4.1)	37 (4.5)	22 (2.7)	36 (3.4)	<0.001
Time to event/censoring [days]	1186.2 ± 285.3	1168.2 ± 299.3	1198.1 ± 271.7	1199.3 ± 276.3	1210.4 ± 274.5	1162.3 ± 297.1	0.760
Age [years]	67.9 ± 9.3	75.4 ± 7.4	70.6 ± 8.5	67.5 ± 8.3	65.2 ± 8.1	61.6 ± 7.6	<0.001
On-treatment DBP [mm Hg]	67.7 ± 8.3	56 ± 3.8	63.2 ± 1.4	67.6 ± 1.1	71.4 ± 1.1	78.3 ± 4.4	<0.001
On-treatment SBP [mm Hg]	120.9 ± 8.6	120.2 ± 8.3	119.6 ± 7.5	119.4 ± 7.7	120.5 ± 7.8	124.2 ± 9.8	<0.001
On-treatment PP [mm Hg]	53.3 ± 10.5	64.2 ± 9.2	56.4 ± 7.7	51.8 ± 7.6	49.1 ± 7.8	45.9 ± 8.7	<0.001
Baseline DBP [mm Hg]	78.2 ± 11.9	66.7 ± 9.2	74.5 ± 8.6	78.8 ± 9.5	82.1 ± 9.5	87.4 ± 10.3	<0.001
Baseline SBP [mm Hg]	139.6 ± 15.7	140.3 ± 14.9	139.6 ± 15.4	139 ± 15.8	138.5 ± 15.9	140 ± 16.4	0.367
Baseline PP [mm Hg]	61.3 ± 14.2	73.6 ± 13.2	65.1 ± 12.2	60.3 ± 11.7	56.5 ± 11.7	52.6 ± 11.7	<0.001
Female (%)	1594 (35.8)	306 (34.5)	296 (34.5)	303 (36.6)	299 (36.1)	390 (37)	0.693
Black race (%)	1382 (31)	179 (20.2)	191 (22.3)	252 (30.5)	286 (34.5)	474 (45)	<0.001
History of cardiovascular disease (%)	737 (16.6)	222 (25.1)	164 (19.1)	128 (15.5)	112 (13.5)	111 (10.5)	<0.001
History of chronic renal disease (%)	1263 (28.4)	359 (40.5)	248 (28.9)	223 (27)	201 (24.3)	232 (22)	<0.001
Body mass index [kg/m^2^]	29.9 ± 5.8	28.3 ± 5.3	29.5 ± 5.2	30.2 ± 6	30.2 ± 5.6	31.3 ± 6.2	<0.001
Current smoking (%)	604 (13.6)	64 (7.2)	83 (9.7)	111 (13.4)	113 (13.6)	233 (22.1)	<0.001
Former smoking (%)	1886 (42.4)	450 (50.8)	392 (45.7)	348 (42.1)	328 (39.6)	368 (34.9)	<0.001
Never smoking (%)	1960 (44)	372 (42)	382 (44.5)	367 (44.4)	387 (46.7)	452 (42.9)	0.329
Number of antihypertensive drugs	1.9 ± 1	2 ± 1.1	1.9 ± 1	1.8 ± 1	1.7 ± 1	1.8 ± 1.1	<0.001
Statin (%)	1896 (42.8)	463 (52.7)	418 (48.8)	356 (43.4)	318 (38.5)	341 (32.5)	<0.001
Aspirin (%)	2302 (51.8)	562 (63.5)	477 (55.7)	443 (53.6)	386 (46.7)	434 (41.3)	<0.001
Cholesterol [mg/dl]	190.3 ± 41.5	181 ± 38.6	185.5 ± 38.9	191.2 ± 41.5	196 ± 44	196.8 ± 42.1	<0.001
HDL [mg/dl]	52.9 ± 14.3	54.1 ± 15	53 ± 13.5	53.3 ± 14.2	52.3 ± 15.2	51.8 ± 13.7	<0.001
Non-HDL [mg/dl]	137.4 ± 39.7	126.9 ± 36.3	132.4 ± 36.9	137.9 ± 38.7	143.6 ± 42.8	145 ± 40.4	<0.001
Triglyceride [mg/dl]	125.3 ± 87	116.7 ± 83.6	120.5 ± 70.4	124.4 ± 70.4	133.3 ± 126.5	130.8 ± 74	<0.001
Glucose [mg/dl]	98.9 ± 13.7	99.2 ± 12.3	99.4 ± 13	99.3 ± 14.7	98.5 ± 13.5	98 ± 14.6	0.022

SBP – systolic blood pressure, DBP – diastolic blood pressure, PP – pulse pressure, p-value calculated for comparison within quintiles.

The highest rates cardiovascular and renal disease and older age were observed among the quintiles with the highest incidence of primary endpoints. Despite having lower SBP, worse prognoses were observed in the quintiles with lower DBP. The patients in quintiles with lower DBP received more antihypertensive drugs. The difference in pulse pressure was also observed between the quintiles in both treatment arms. The patients with lower on-treatment DBP had also lower baseline DBP. Higher use of statins and aspirin was associated with better lipid profile, but this result was observed in the quintiles with worse outcomes. Similar findings were observed in both treatment arms.

The primary outcome rates were compared between the corresponding DBP quintiles in standard and intensive treatment arm. When the 1^st^ and 2^nd^ quintiles were compared, there was a higher incidence of primary outcomes in the standard treatment arm compared to the intensive treatment arm (10.8% vs 6.7%, p = 0.003 and 6.7% vs 4.1%, p = 0.023). There was no significant difference in between the 3^rd^, 4^th^ and 5^th^ DBP quintiles in the two treatment arms (5.1% vs 4.5%, p = 0.649; 4.4% vs 2.7%, p = 0.064, 4.3% vs 3.4%, p = 0.342).

**Figure 3:**
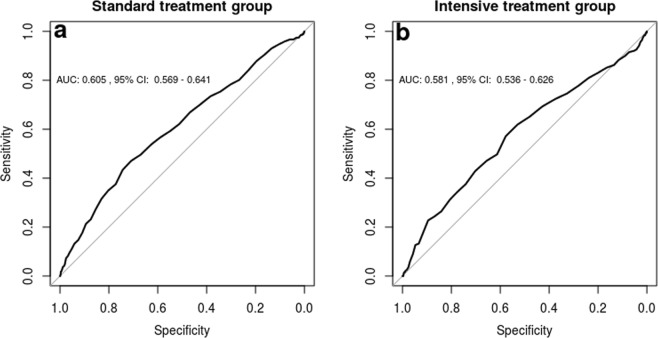
Receiver operating curves of DBP among the standard (**a**) and intensive (**b**) treatment arms with computed area under the curve (AUC) and 95% confidence intervals.

Among the standard and intensive treatment arms, on-treatment DBP was a poor predictor of primary endpoints. Receiver operating characteristic (ROC) analysis showed the following area under the curve (AUC) measurements: 0.605 (95% CI: 0.569–0.641) and 0.581, 95% CI: 0.536–0.626 (Fig. 3).

We used Cox proportional hazard risk model to assess the impact of on-treatment DBP and pulse pressure on primary endpoint event risks among the total analysed population of patients. After adjusting for age, sex, history of clinical cardiovascular disease or chronic kidney disease, treatment arm, current smoking status and body mass index, our analysis revealed no influence of on-treatment DBP on the occurrence of primary outcome (Table 3). However, on-treatment pulse pressure was shown to be an independent predictor of primary outcome event risks after adjusting for other cofactors in the multivariable Cox model (Table 3). The analysis comparing participants in the quintiles of total analysed population is shown in Table S1 and Fig. S1.

**Table 3:** Cox proportional hazard risk model evaluating the impact of on-treatment DBP (Model A) or on-treatment pulse pressure (Model B) and other predictors on primary outcome occurrence.

Parameter	Hazard risk	95% confidence interval	p-value
**Model A**			
Age	1.04	1.03–1.06	<0.001
Female sex	0.75	0.61–0.92	0.006
History of cardiovascular disease	2.26	1.85–2.75	<0.001
History of chronic kidney disease	1.41	1.16–1.72	<0.001
Allocation to the intensive treatment arm	0.63	0.51–0.76	<0.001
On-treatment DBP	0.99	0.98–1.0	0.159
Current smoking status	2.08	1.59–2.71	<0.001
BMI	1.02	1.0–1.03	0.054
**Model B**			
Age	1.041	1.03–1.05	<0.001
Female sex	0.747	0.61–0.92	0.005
History of cardiovascular disease	2.26	1.85–2.76	<0.001
History of chronic kidney disease	1.393	1.14–1.7	0.001
Allocation to the intensive treatment arm	0.723	0.59–0.88	0.001
Current smoking status	2.066	1.58–2.7	<0.001
On-treatment pulse pressure	1.01	1–1.02	0.039
BMI	1.019	1–1.04	0.034

BMI – body mass index, DBP – diastolic blood pressure.

## Discussion

This study shows that DBP is not an independent risk factor for cardiovascular events. Worse prognoses among participants with low on-treatment DBP may be explained by the fact that these patients were older, often had previous cardiovascular disease or chronic kidney disease and more often were smokers. The impact of those factors on the primary endpoints was high, what is expressed by hazard ratios in Table 3.

Three other studies based on SPRINT data investigated the J-curve hypothesis of DBP. Kalkman *et al*. re-analysed merged SPRINT and ACCORD data to verify the J-shaped curve hypothesis related to SBP and DBP^13^. The authors found an increased risk associated with lower on-treatment DBP with a nadir at 85 mmHg, which indicated that their results confirmed J-shaped curve hypothesis. However, the impact of potential interactions between low DBP and confounding variables was not assessed. In our study, the influence of low on-treatment DBP on primary outcomes disappeared after adjusting for confounding factors.

In another paper re-analysing the SPRINT data, Stensrud *et al*. assumed, according to the results of previous studies, that mean DBP < 60 mmHg may worsen the effects of lowering BP^14,18^. After adjustment for potential confounding variables, the authors did not observe unfavourable effects of DBP < 60 mmHg. It is important to note that the authors used a cut-off value of DBP < 60 mmHg while in other studies, DBP < 70 mmHg was used as the lower limit for DBP^19–21^. These results may imply that DBP < 60 mmHg did not increase patients’ risk when cofactors were included in the analysis. Also, recent European guidelines selected DBP = 70 mmHg as a lower limit for recommended on-treatment DBP^3^. The role of pulse pressure should be also emphasised here. We found that on-treatment pulse pressure is an independent risk factor for primary endpoint events, even after adjusting for confounding factors. In the study examining SPRINT data, Pareek *et al*. showed that the excess risk associated with higher pulse pressure at baseline appeared to be related to age, sex and other major cardiovascular risk factors, thus limiting its clinical utility among high-risk SPRINT patients^22^. Authors concluded that baseline pulse pressure should not limit the selection of appropriate candidates for intensive BP reduction. However, some studies found that baseline pulse pressure might be an independent risk factor for acute coronary events^23^.

The SPRINT data analysis was also performed by Lee *et al*.^15^. The authors determined that DBP < 55 mmHg at during a single visit was associated with adverse clinical effects. The adopted methodology evaluated the effect of single episode of hypotension rather than examining the effect of permanently low on-treatment DBP on adverse events. In contrast to the results of a study by Stensrud *et al*., DBP < 55 mm at a single visit significantly increased the risk of complications according to the results of a multivariable Cox proportional analysis.

According to the results of previous SPRINT re-analyses, our study involved the comparison within the quintiles of DBP in both treatment arms. This methodology helped reveal the key differences within subpopulations across on-treatment DBP levels and the necessity of assessing the impact of confounding factors.

The results of other studies investigating the DBP-related J-curve hypothesis were ambiguous. The differences between analysed endpoints, DBP thresholds and study populations (baseline prevalence of CAD, diabetes mellitus or stroke) made it difficult to draw clear conclusions and compare results with our results.

The results of HOT trial (Hypertension Optimal Treatment) did not reveal differences in the incidence of stroke risk, overall and cardiovascular-related mortality and myocardial infarction between the subgroups with DBP ≤90, ≤85 and ≤80 mmHg. The incidence of all myocardial infarctions was almost significant, and the authors concluded that optimal DBP is 82.6 mmHg^10^. In the Hypertension Objective Treatment Based on Measurement by Electrical Devices study (HOMED-BP), the impact of usual (125–134/80–84 mmHg) vs. tight (<125/80 mmHg) BP control was investigated^24^. The authors did not evidence regarding a J-shaped curve relationship between BP reduction and clinical endpoints^24^. According to the J-shaped curve hypothesis, low compliance among the tight control arm, on-treatment DBP may be higher than unsafe BP levels. Post-hoc analyses of The Valsartan Antihypertensive Long-Term Use Evaluation and INVEST trials did not provide data on the detrimental effects of low on-treatment DBP^11,25^. Other re-analyses confirmed the J-shaped hypothesis for on-treatment DBP in subjects of Ongoing Telmisartan Alone and in combination with Ramipril Global Endpoint Trial (ONTARGET) and merged ONTARGET and Telmisartan Randomised Assessment Study in ACE Intolerant Participants with Cardiovascular Disease (TRANSCEND) trials^12,19,26^. The J-shaped curve relationship between DBP and cardiovascular mortality was also observed after long-term follow-up in the analysis of the National Health and Nutrition Examination Survey III and Diabetes Heart Study performed by Gomadam *et al*.^27^ Our multivariable Cox model showed that DBP was not a significant cofactor. Based on the data obtained from this study and previous studies, the evidence for a J-shaped relationship between DBP and cardiovascular risk is inconclusive. Despite the weakness of evidence regarding the J-shaped curve hypothesis, this phenomenon is more pronounced in the subjects with CAD^28,29^. Furthermore, CAD is more frequent in the subjects with lower DBP, which suggests that this effect may be cumulative^20,30–32^. In our analysis of individuals with a high risk of cardiovascular disease, including current CAD patients, we found no evidence that a J-shaped curve existed for on-treatment DBP when some cofactors were included into the analysis.

Despite the fact that coronary blood flow primarily occurs during diastole and depends on DBP, it should be emphasised that the clinical effects of DBP cannot be interpreted separately from on-treatment SBP. It is difficult to decide on a patient-by-patient basis whether the benefit of intensive SBP reduction outweighs the harms associated with low DBP. Beddhu *et al*. evaluated the interaction between low baseline DBP and intensive SBP reduction among SPRINT participants and found adverse effects associated with intensive treatment to lower SBP among patients with low baseline DBP^33^. Our comparison of corresponding quintiles of DBP in both treatment arms suggests better outcomes are associated with intensive rather than standard treatment, despite lower on-treatment DBP. According to these results, on-treatment DBP should be only interpreted as the consequence of SBP lowering and SBP < 130 mmHg should be considered a treatment goal.

The most important limitation to our study is the fact that despite the large sample size, post-hoc reasoning could be affected by potential bias. Secondly, there were essential differences identified between automated and clinical BP measurements. Therefore, it was difficult to compare our results to those of the SPRINT trial due to differences in methods^34,35^. The studies performed by Kalkman, Stensrud and Lee had the same disadvantage; therefore, their choice of corresponding harmful DBP threshold may be also questioned. Furthermore, our findings do not apply to diabetic subjects or these with the history of stroke. However, the ACCORD and SPS3 trials already investigated these subgroups^17,18^. Considering the results of Di Nora *et al*., who showed that the SPRINT results were not reproducible within a community-based cohort of Caucasian participants, our results could also vary based on the characteristics of the investigated population^36^.

In conclusion, our data showed that low on treatment DBP does not independently influence cardiovascular risk in patients allocated to intensive and standard blood pressure lowering strategies. These results are in line with the conclusions of similar studies^11–14,27^. In that context, low on-treatment DBP should be considered as a marker of high cardiovascular risk rather than an independent risk factor^26^.

## Methods

### Study population

SPRINT was a randomised and multi-centre study that determined whether patients benefitted from intensive efforts to decrease SBP^1^. Participants were randomised to intensive (target SBP < 120 mmHg) and standard treatment (target SBP < 140 mmHg) arms. Although a target of DBP was not established, patients were treated to achieve DBP of <90 mmHg after meeting their goal SBP measurement^37^. Nearly 10,000 subjects who presented high risks of cardiovascular diseases and adverse cardiovascular events (older age; SBP, 130–180 mmHg and the history of cardiovascular or chronic kidney disease or Framingham Risk Score for 10-year cardiovascular risk >15%) were enrolled in the study. Eligibility and exclusion criteria are described previously^38^.

### BP measurements and other clinical data

SBP and DBP were measured three times per visit using an automated system (Model 907, Omron Healthcare). According to the study protocol, we decided to analyse the interval from the 6-month visit until the end of the study due to the stability of BP values among the participants. We used the mean of BP measurements on each visit during the analysed period to describe on-treatment DBP and SBP. The pulse pressure was calculated as the difference between SBP and DBP. Other data including age, body mass index, height, lipid profile, blood glucose levels and overall medical history were considered.

### Outcome

The primary composite outcomes of the SPRINT trial included myocardial infarction, acute coronary syndrome not resulting in myocardial infarction, stroke, acute decompensated heart failure or death from cardiovascular complications. SPRINT proved that allocation to the intensive treatment arm improved cardiovascular outcomes among hypertensive patients with a high risk of cardiovascular disease. We decided to investigate the association between DBP and primary outcomes, as defined by the original trial.

### Data source

Anonymised data (SPRINT_ POP Research Materials) for the current analysis were obtained from the National Heart, Lung and Blood Institute (NHLBI) Biologic Specimen and Data Repository Information Coordinating Centre. This manuscript does not necessarily reflect the opinions or views of the SPRINT_POP or the NHLBI. Our study received the approval of the Ethics Committee at Medical University of Warsaw. The SPRINT study was approved by the institutional review board at each participating study site. Informed consent was obtained from all participants. All methods were carried out in accordance with relevant guidelines and regulations.

### Statistical analysis

This was a retrospective study. The investigations were performed within the subgroups selected by DBP quintiles separately for the subjects assigned to intensive and standard treatment arms. Continuous data were presented as mean and standard deviation. Discrete values were presented as number and percentage. The analysis of variance and Chi Square (χ^2^) tests were used for between-arm comparisons. Survival was analysed using Kaplan–Meier curves, and log-rank test was applied to assess difference in survival curves. ROC curve analysis was used to assess the predictive value of DBP. A Cox proportional hazard risk model was used to explain the impact of the independent variable (DBP) and other predictors on the occurrence of primary composite outcomes. A p-value < 0.05 was considered significant. All employed tests were two-tailed. All calculations were made using R 3.4.0 software (R Foundation for Statistical Computing, Vienna, Austria) for statistical computations. Standard, ‘survival’, ‘survminer’ and ‘pROC’ packages were used^39–42^.

## Supplementary information

Supplementary Material

## Data Availability

Data that support the findings of this study are available from the NHLBI, but restrictions apply regarding the availability of these data. Since these data are under the licence for the current study, they are not publicly available. The data are available from NHLBI upon reasonable request. The authors have no right to share the data.
