# Supplementary material for: Low on-treatment diastolic blood pressure and cardiovascular outcome: A post-hoc analysis using NHLBI SPRINT Research Materials
Source: Sci Rep. 2019 Sep 10;9:13070. doi: 10.1038/s41598-019-49557-4 (PMC6737094; doi:10.1038/s41598-019-49557-4)
Supplement: Supplementary file 1 — Supplementary Material [file 41598_2019_49557_MOESM1_ESM.pdf]

Low on-treatment diastolic blood pressure and cardiovascular outcome. A post-hoc analysis using NHLBI SPRINT Research Materials

Authors: Piotr Sobieraj[1*] MD; Jacek Lewandowski[1] MD, PhD; Maciej Siński MD[1], PhD; Zbigniew Gaciong[1] Professor, MD, PhD.

[1]Department of Internal Medicine, Hypertension and Vascular Diseases, The Medical University of Warsaw, Warsaw, 02-091, Poland

[*] piotr.sobieraj@wum.edu.pl

Parameter	1st quintile 38-63 mm Hg N=1602	2nd quintile 63-69 mm Hg N=1788	3rd quintile 69-74 mm Hg N=1870	4th quintile 74-80 mm Hg N=1804	5th quintile 80-113 mm Hg N=1826	p-value
Primary endpoint event (%)	132 (7.4)	104 (5.8)	74 (4.2)	69 (3.9)	82 (4.6)	<0.001
Time to event/censoring [days]	1163.7±299.8	1180.4±279.6	1191.2±281.8	1201.8±273.8	1164.7±292.8	0.275
Age [years]	75.2±7.8	70.2±8.5	67.6±8.7	64.9±8.2	61.3±7.2	<0.001
On-treatment DBP [mm Hg]	58.1±4.1	66.2±1.7	71.3±1.4	76.5±1.7	84.8±4.1	<0.001
On-treatment SBP [mm Hg]	123.8±10.4	124.4±10.3	126.9±10.4	130±9.6	135.5±8.3	<0.001
On-treatment PP [mm Hg]	65.7±11	58.1±10.3	55.6±10.3	53.5±9.5	50.8±7.8	<0.001
Baseline DBP [mm Hg]	67.3±9.5	74.9±9.4	78.6±9.7	82.3±9.7	87.6±10.2	<0.001
Baseline SBP [mm Hg]	140.5±15.5	139.3±15.4	138.7±15.5	139.5±15.8	140±15.5	0.46
Baseline PP [mm Hg]	73.3±14.1	64.5±12.5	60±12.2	57.2±12	52.4±10.7	<0.001
Female (%)	643 (36.2)	648 (36.4)	643 (36.1)	651 (36.6)	554 (31.2)	0.002
Black race (%)	349 (19.7)	445 (25)	529 (29.7)	630 (35.4)	823 (46.3)	<0.001
History of cardiovascular disease (%)	424 (23.9)	359 (20.2)	281 (15.8)	225 (12.6)	181 (10.2)	<0.001
History of chronic renal disease (%)	696 (39.2)	543 (30.5)	469 (26.3)	426 (23.9)	357 (20.1)	<0.001
Body mass index [kg/m2]	28.2±5.2	29.5±5.5	29.8±5.7	30.6±6	31.2±5.9	<0.001
Current smoking (%)	114 (6.4)	197 (11.1)	198 (11.1)	282 (15.9)	379 (21.3)	<0.001
Former smoking (%)	884 (49.8)	822 (46.2)	763 (42.8)	685 (38.5)	633 (35.6)	<0.001
Never smoking (%)	774 (43.6)	757 (42.6)	820 (46)	811 (45.6)	763 (42.9)	0.128
Number of antihypertensive drugs	2±1	1.8±1	1.8±1	1.7±1	1.8±1.1	<0.001
Statin (%)	938 (53.2)	856 (48.5)	787 (44.4)	694 (39.2)	593 (33.6)	<0.001
Aspirin (%)	1082 (61.1)	994 (56)	902 (50.7)	836 (47.1)	732 (41.3)	<0.001
Cholesterol [mg/dl]	181.2±38.5	187.2±40.4	191.6±42.9	193.7±41.4	196.6±40.1	<0.001
HDL [mg/dl]	53.9±14.4	53.6±14.4	53.1±14.9	52.3±14.6	51.4±13.6	<0.001
Non-HDL [mg/dl]	127.4±36.4	133.6±37.8	138.6±41.1	141.5±39	145.2±38.4	<0.001

Triglyceride [mg/dl]	117.7±75.3	122.9±103.6	126.2±101.8	129.2±76.2	134.5±92	<0.001
Glucose [mg/dl]	99.3±12.4	99.3±13.7	98.3±12.7	98.6±12.6	98.5±15.6	0.022

Table S1. Characteristic of DBP quintiles within both standard and intensive treatment arm. Continuous data are presented as mean ± standard deviation, discrete as a number with percentage. SBP – systolic blood pressure, DBP – diastolic blood pressure, PP – pulse pressure, p-value calculated for comparison within quintiles.

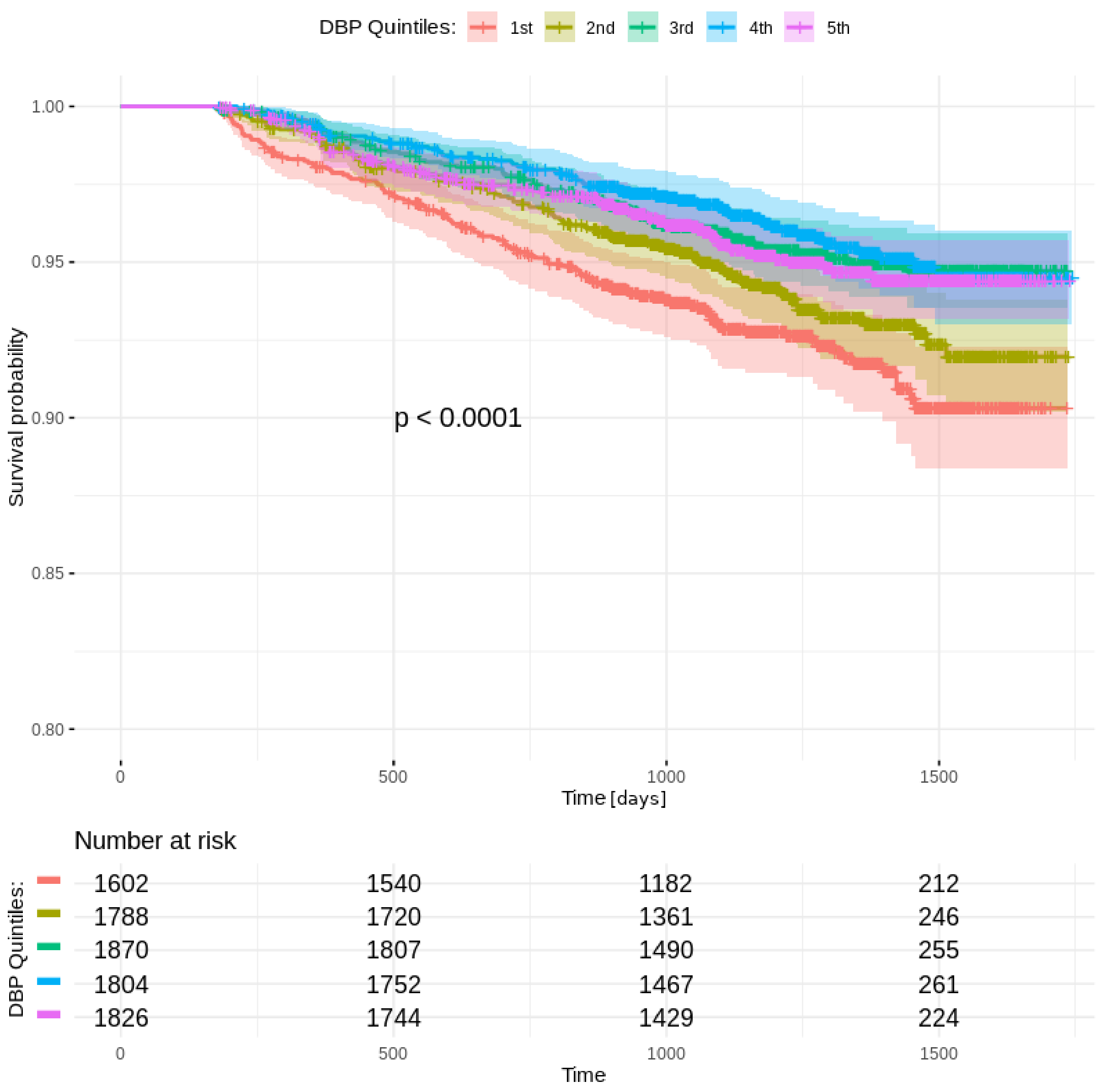

Figure S1. Kaplan-Meier curves with 95% confidence intervals in both standard and intensive treatment arm. Comparison of curves within DBP quintiles were made using the log-rank test (p<0.0001). 1st quintile: 38-63 mmHg, 2nd quintile: 63-69 mmHg, 3rd quintile: 69-74 mmHg, 4th quintile 74-80 mmHg, 5th quintile 80-113 mmHg.
